# Pathoblockers or antivirulence drugs as a new option for the treatment of bacterial infections

**DOI:** 10.3762/bjoc.14.239

**Published:** 2018-10-11

**Authors:** Matthew B Calvert, Varsha R Jumde, Alexander Titz

**Affiliations:** 1 Chemical Biology of Carbohydrates, Helmholtz Institute for Pharmaceutical Research Saarland (HIPS), Helmholtz Centre for Infection Research (HZI), D-66123 Saarbrücken, Germany; 2 Deutsches Zentrum für Infektionsforschung (DZIF), Standort Hannover-Braunschweig, Germany; 3 Department of Pharmacy, Saarland University, Saarbrücken, Germany

**Keywords:** antimicrobial resistance, bacterial adhesins, bacterial toxins, pathoblockers, quorum sensing

## Abstract

The rapid development of antimicrobial resistance is threatening mankind to such an extent that the World Health Organization expects more deaths from infections than from cancer in 2050 if current trends continue. To avoid this scenario, new classes of anti-infectives must urgently be developed. Antibiotics with new modes of action are needed, but other concepts are also currently being pursued. Targeting bacterial virulence as a means of blocking pathogenicity is a promising new strategy for disarming pathogens. Furthermore, it is believed that this new approach is less susceptible towards resistance development. In this review, recent examples of anti-infective compounds acting on several types of bacterial targets, e.g., adhesins, toxins and bacterial communication, are described.

## Review

### 1. Antimicrobial resistance crisis for bacterial infections

The current crisis caused by antimicrobial resistance [1–2] demands new strategies to fight infections. Antibiotics have served as life-saving drugs during the last 100 years and rescued the world from a situation where practically untreatable infections with high mortality rates were the norm. However, starting in the 1960s, the delusive belief that the available antibiotics were sufficiently effective to treat all infections led to a decline in the development of new antibiotics, with very few new antibiotics addressing a novel mode of action being brought to the market over the last four decades [3].

In parallel with the decline of new antibiotics, resistance towards these widely used drugs has evolved at a high pace and multi as well as extreme drug resistant (MDR/XDR) strains of pathogens are now commonplace. Exposure of bacteria to compounds directly acting on bacterial viability, such as antibiotics, intrinsically leads to the development of resistance as a matter of microbial survival. This so-called selection pressure can lead to the overgrowth of the initial infective population with a resistant variant of the pathogen, rendering the antibiotic substance ineffective. Especially prevalent in the hospital setting, the abundance of resistance prevents efficient treatment of infected patients. The so-called ESKAPE pathogens, [4] *Enterococcus faecium, Staphylococcus aureus, Klebsiella pneumoniae, Acinetobacter baumanii, Pseudomonas aeruginosa,* and *Enterobacter* species, were initially identified as the most problematic ones. In 2017, an extended list of twelve pathogens, currently considered as those with the highest importance, was published by the WHO [5]. Emphasizing the current crisis, in 2017 one report described a patient infected with a pan-resistant *Klebsiella* strain, where no available drug was efficacious and the patient finally died from septic shock [6]. Therefore, new antibiotics and new alternative treatments are urgently needed.

### 2. Concept of antivirulence drugs or pathoblockers

Bacterial virulence is the prime determinant for the deterioration of an infected patient’s health. Blocking bacterial virulence, or pathogenicity, is a new approach that has emerged over the last decade [7–9]. The pubmed.gov database yields 292 references on the topic (as of 06/08/2018), with an exponential increase over the years. Unfortunately, as the terms ‘antivirulence’ and ‘pathoblocker’ are often used interchangeably, many publications in the field are not found in this type of search, for example the pioneering review by Clatworthy et al. in 2007, entitled ‘Targeting virulence: a new paradigm for antimicrobial therapy’ [8], which has been cited approximately 800 times.

In sharp contrast to traditional antibiotics that kill or impair bacterial viability, this new approach aims to disarm the pathogen. Interfering with the interaction of the pathogen with its host in this way is believed to both reduce damage to the host and to enable the host to clear the microbe from its system. Furthermore, as antivirulence drugs do not kill, it is believed that the selection pressure for resistant mutants will be significantly reduced. In some cases, however, resistance has already been observed (e.g., through increased expression of efflux pumps to circumvent quorum quenching), with the likelihood of the appearance of resistance mechanisms seemingly dependent upon the importance of the targeted virulence factor to the pathogen [10].

### 3. Blocking adhesion and biofilm formation

Bacterial adhesion to the host’s tissue is the initial step of every infection. In many cases, microbial adhesion is mediated by carbohydrate-binding proteins, so-called lectins, which recognize glycoconjugates on the surface of cells and tissue. Surface exposed glycoconjugates are highly abundant on all living cells and are generally referred to as the glycocalyx. Bacterial lectins act as adhesins with defined carbohydrate-binding specificities, in order to establish and maintain infection of the host’s various tissues and organs. Therefore, the inhibition of this adhesion process using glycomimetics as pathoblockers has developed as an area of active research in the last two decades [11–12].

Uropathogenic *Escherichia coli* (UPEC) is a major cause for chronic and recurrent urinary tract infections. These bacteria employ lectins in order to attach to and invade bladder and kidney tissue, and to promote biofilm formation. Bladder-adhesive FimH is a mannose-specific lectin and the kidney-adhesive PapG binds galactosides. In a second indication, FimH also mediates the attachment of *E. coli* to the gut, inducing inflammation in Crohn’s disease [13]. The crystal structure of FimH was published by Hultgren and Knight et al. in 1999 [14]. FimH is highly specific for α-D-mannoside ligands with this residue residing in a carbohydrate binding pocket with its α-linked substituent towards an adjacent cleft. This substituent, termed the aglycon, can also interact with the tyrosine gate formed by Tyr48 and Tyr137 [15–16], as well as form hydrogen bonds and electrostatic interactions with the Arg98/Glu50 salt bridge of the protein. Taking this coordination geometry into consideration for further ligand optimization, it was found important to focus on the aglycon part of the mannosides.

**Figure 1:**
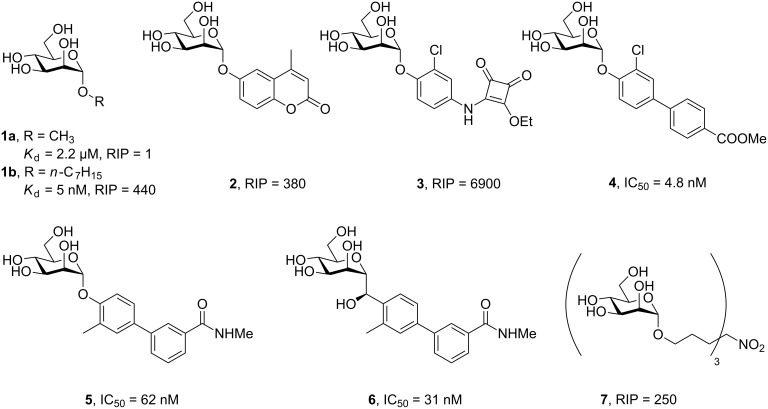
Mannosides as inhibitors of the lectin FimH from uropathogenic *Escherichia coli*.

The attachment of lipophilic aglycons to an α-linked mannose residue was identified to increase the binding potency tremendously due to the opening of a lipophilic cleft on FimH, the tyrosine gate [15]. Various alkyl mannosides **1** (Figure 1) were analyzed and *n*-heptyl mannoside (**1b**) revealed the highest potency, as a result of it having the optimal length to bind to the tyrosine gate. Lindhorst and co-workers have demonstrated that mannosides with an extended aromatic aglycon could further improve the interaction as shown for compounds **2** and **3**. Their relative inhibitory potential (RIP), which is benchmarked with the reference methyl α-D-mannoside (**1a**) defined as RIP = 1, was increased up to 6900-fold [17].

The biphenyl mannosides (e.g., **4**, **5**) have subsequently been identified by the Ernst and Hultgren/Janetka groups as promising inhibitors of FimH-mediated bacterial adhesion in mice [18–19]. These compounds have been extensively optimized in many works published by both groups, culminating in the identification of mannophosphates as prodrugs to increase oral bioavailability [20] and mannose *C*-glycosides, such as compound **6** , demonstrating enhanced in vivo metabolic stability [21]. Such biphenyl mannoside-derived compounds are the current state of the art and are being further developed by the company Fimbrion (St Louis, MO) in collaboration with GlaxoSmithKline.

In many cases, lectins have more than one carbohydrate binding site or are clustered in proximity. Therefore, multivalent display of lectin and ligand results in a higher avidity [22–23]. The Lindhorst group also synthesized and analyzed so-called glycoclusters, e.g., **7** (Figure 1), where the saccharide moiety is displayed in a multivalent fashion [17,24–26]. When this simple trimannosylated compound was tested in a whole cell ELISA, it was shown that the apparent binding affinity increases by a factor of 250 versus methyl α-D-mannoside, while the valency only increased by a factor three.

It should be noted that the full length FimH adhesin consists of two domains, a lectin and a pilin domain that are interconnected by a hinge region. Interestingly, in vitro binding studies have been performed with the lectin domain only. Recent works suggested that the conformation of the two domains influence the protein’s affinity towards inhibitors and the biologically relevant state is a matter of ongoing research [27–28].

**Figure 2:**
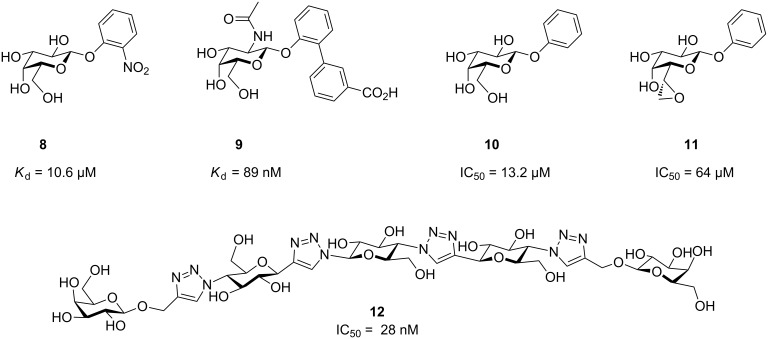
Galactosides targeting uropathogenic *Escherichia coli* FmlH (compounds **8** and **9**) and *Pseudomonas aeruginosa* LecA (compounds **10**–**12**).

Another adhesin of uropathogenic *E. coli* is FmlH, which is located at the tip of F9 pili and binds β-D-galactosides with moderate potency. It could be shown that this lectin plays an important role in kidney-associated chronic UTIs, as its glycan receptor is abundantly expressed in this organ. In screening assays, 2-nitrophenyl galactoside (**8**) was identified displaying a dissociation constant of 10.6 µM (Figure 2). A detailed optimization program run by the Hultgren and Janetka groups yielded derivatives of *N*-acetyl galactosamine bearing biphenyl aglycons, such as compound **9**, as very potent ligands of this protein. Beyond blocking the binding site of FmlH on the pili of *E. coli*, these compounds proved effective at promoting eradication of bacteria from murine kidney in synergy with a mannoside for FimH [29].

*P. aeruginosa* is one of the highly resistant ESKAPE pathogens that, in addition to antimicrobial resistance, forms biofilms, a complex matrix of extracellular polysaccharides, polypeptides and DNA, which act as an additional protective barrier [30]. *P. aeruginosa* employs two lectins for biofilm formation and host–cell adhesion: proteins LecA and LecB [31–32] which are also important for mediating bacterial virulence in vivo [33]. Therefore, both LecA and LecB have served as targets for pathoblocker development [22–23,30,34–35]. As a result of the comparatively low affinity of both lectins towards their natural carbohydrate ligands (α-galactosides for LecA and α-fucosides and mannosides for LecB), numerous multivalent presentations have been developed with the aim to improve affinity based on avidity [22].

LecA recognizes aryl β-D-galactosides with moderate potency, e.g., compound **10** (Figure 2). However, attempts to optimize the potency by varying the aryl substitution resulted in a flat SAR with only little variation in potency among the substituents analyzed [30,36–39]. Just recently, in an attempt to search for new pharmacophores, Titz et al. have reported the synthesis of the epoxyheptose derivative **11** targeting a cysteine residue of LecA with its electrophilic epoxide warhead [40]. It could be demonstrated that **11** is a covalent lectin inhibitor, which provided the first proof-of-concept for this new approach to lectin inhibition. To date, the most potent LecA inhibitor **12** has been designed by the Pieters group, where two galactoside moieties are optimally oriented in space to simultaneously bind to two of the four binding sites in LecA [41]. This optimal geometric match to LecA resulted in low nanomolar inhibition of LecA.

**Figure 3:**
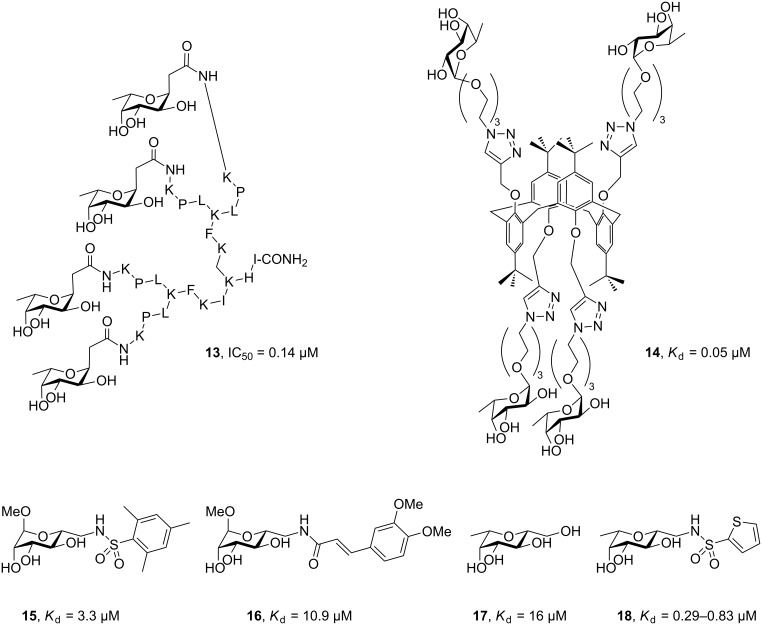
Mannosides and fucosides as inhibitors of *P. aeruginosa* LecB.

LecB has been studied in detail using multivalent and small molecule approaches. Interestingly, the sequence of LecB differs among clinical isolates of this highly variable pathogen, with some mutations in close proximity to the carbohydrate binding site, but carbohydrate-binding function is preserved across all lectins investigated [42–43]. The glycopeptide dendrimer **13** (Figure 3) showed potent inhibition of biofilm formation and synergistically acted with tobramycin to eradicate biofilm-embedded bacteria in vitro [44–45]. Also, the fucosylated tetravalent calixarene **14** proved a potent ligand to LecB (*K*_d_ = 48 nM) and showed beneficial effects in an acute murine pulmonary infection model following inhalative administration [46]. Despite its LecB-mediated in vivo activity, this compound had no effect on biofilms in vitro at concentrations up to 2000-fold above the *K*_d_; a biofilm reduction by 80% could be achieved at concentrations as high as 100000-fold above *K*_d_ (5 mM). For a future systemic application, Titz et al. have developed small molecule LecB inhibitors derived from mannose and obtained potent monovalent inhibitors (compound **15**) of LecB-mediated bacterial adhesion [47]. The sulfonamide **15** and cinnamide **16** were developed to take advantage of interactions with a nearby shallow pocket, and indeed these compounds showed superior thermodynamics and kinetics of binding to LecB compared to mannose, resulting in a prolonged receptor residence time of several minutes [48]. In a complementary approach, glycomimetic *C*-glycoside **17** was obtained, aiming at improved metabolic stability and selectivity [49]. Both approaches were then combined into low molecular weight *C*-glycosidic sulfonamides, which resulted in very potent LecB and *P. aeruginosa* biofilm inhibitors with over 80% inhibition at a concentration of 100 µM [50]. Compound **18** of this series further showed very good in vitro stability against plasma and liver microsomes, absence of cytotoxicity, and excellent oral bioavailability in mice.

### 4. Direct toxin inhibition

Numerous bacteria secrete toxins that are responsible for acute virulence. Various small molecule and antibody approaches target the inhibition of bacterial toxins in order to antagonize bacterial virulence [51].

AB toxins are widespread among species and consist of a catalytically active A-domain and one or more units of a receptor-binding domain B. The B domain is responsible for binding to a cell-surface receptor, which engages in receptor-mediated cellular uptake. The AB toxin then migrates either via a classical endocytosis pathway, or via retrograde transport through the secretory pathway into the cytosol, where the A domain can exert its toxic property. AB_5_ toxins are abundant in many pathogens and the B domain is a carbohydrate-binding domain for cell-surface binding. Numerous inhibitors have been developed against AB toxins, targeting toxin transcription, assembly, receptor binding and enzyme function [51].

A set of antibodies against diverse toxins has recently been approved for therapeutic use, which demonstrates the scientific and medical feasibility of entering the market with an antivirulence drug. The monoclonal antibody bezlotoxumab binds to *C. difficile* toxin B and was approved for the prevention of infections with this intestinal pathogen in 2016 [52]. Obiltoxaximab [53] and raxibacumab [54] are two approved antibody treatments for inhalative anthrax that target the *Bacillus anthracis* toxin. It is likely that small molecules will also benefit from the knowhow obtained during the antibody-related clinical studies and it is probably only a matter of time before a small molecule drug is approved.

**Figure 4:**
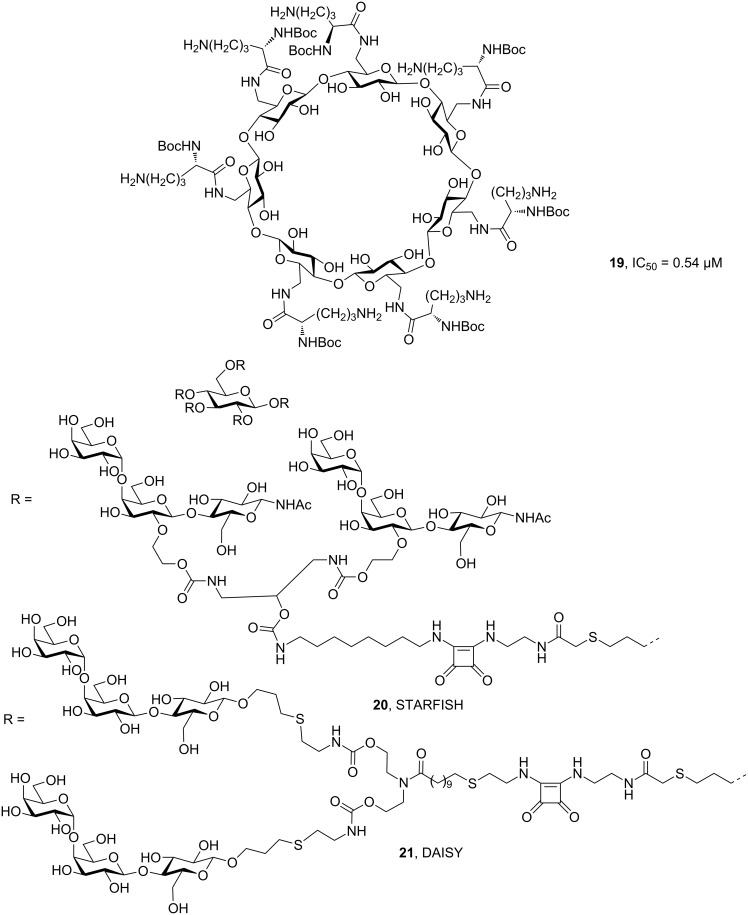
β-Cyclodextrin-based antitoxin **19** against *S. aureus* α-hemolysin and the decavalent Shiga toxin inhibitors STARFISH (**20**) and DAISY (**21**).

Pore forming toxins constitute another large set of virulence factors playing crucial roles in acute virulence [55]. *Staphylococcus aureus* infections are characterized by the toxic action of bacterial α-hemolysin, a pore forming toxin leading to hemolysis. The antibody MEDI4893, which blocks *S. aureus* α-hemolysin, is currently in phase II clinical trials [56]. Despite the challenges associated with the large size of the pore structures, small molecules have also been widely studied as antitoxins and there are examples at various stages of the discovery process [51]. An important example of this concept is the application of cyclodextrins as anti-infectives [57], with the ornithine-substituted compound **19** (Figure 4) being shown to be able to block various pore-forming toxins, as well as successfully preventing and treating infections by *S. aureus* in mice [58].

Enterohemorragic *E. coli* (EHEC) bacteria produce Shiga toxins Stx1 and Stx2 that belong to the group of AB_5_ toxins. These Shiga toxins are the causative agents for bacterial virulence in the gut of the infected host and bind to the P blood group antigens that bear terminal Gal-α-1,4-Gal disaccharides. Blocking Stx1 with decavalent molecules STARFISH (**20**) and Stx1 and Stx2 with DAISY (**21**) resulted in a full protection of mice from the toxin [59–60]. Another set of compounds called SUPER TWIG bears P blood group antigens on the antennae of a carbosilane dendrimer and was developed as an intravenously applied scavenger of circulating Shiga toxins to prevent the most severe complications in these infections [61].

While not typically classed as toxins, bacterial proteolytic enzymes, such as collagenases or elastases, often account for host cell damage and immune evasion. Janda and co-workers developed thiol-based small molecules targeting the active site zinc ion in *P. aeruginosa* elastase LasB showing prolonged survival in a *C. elegans* infection model [62]. Hydroxamic acid-containing molecules addressing the same enzyme were developed by the Hartmann group; these compounds showed a moderate reduction of biofilm formation resulting from a lowered release of the structural biofilm component extracellular DNA [63]. Recently, inhibitors of the clostridial collagenase were discovered that showed high selectivity for the bacterial enzyme over related host metalloproteases [64]. It is hoped that continued research in this area will lead to a complementary class of antivirulence drugs against *Clostridium difficile*, adding to the existing repertoire of clostridial AB antitoxins discussed previously.

### 5. Toxin secretion

A complementary approach to toxin inhibition is the interference with the ability of the bacterium to release the toxin into its environment, i.e., toxin secretion. Many different secretion systems exist in bacteria [65] and the Gram-negative specific type III secretion system (TTSS) is a focus of current research. TTSS is a major virulence determinant in a number of pathogens, including *P. aeruginosa*. In TTSS, toxins are secreted from the bacterial cytosol across the bacterial membranes and the extracellular environment through a needle-like structure into a host cell. The blockade of toxin secretion or needle assembly has been an active area of research, and small molecules as well as antibodies are currently being developed [30,66–67]. The TTSS needle tip protein PcrV was found to be a suitable target to prevent toxin secretion. The anti-PcrV antibody KB001 [68] and the bifunctional antibody MEDI3902 [69], which targets PcrV and the biofilm-associated exopolysaccharide psl, are both currently in phase II clinical trials.

### 6. Bacterial communication

**Figure 5:**
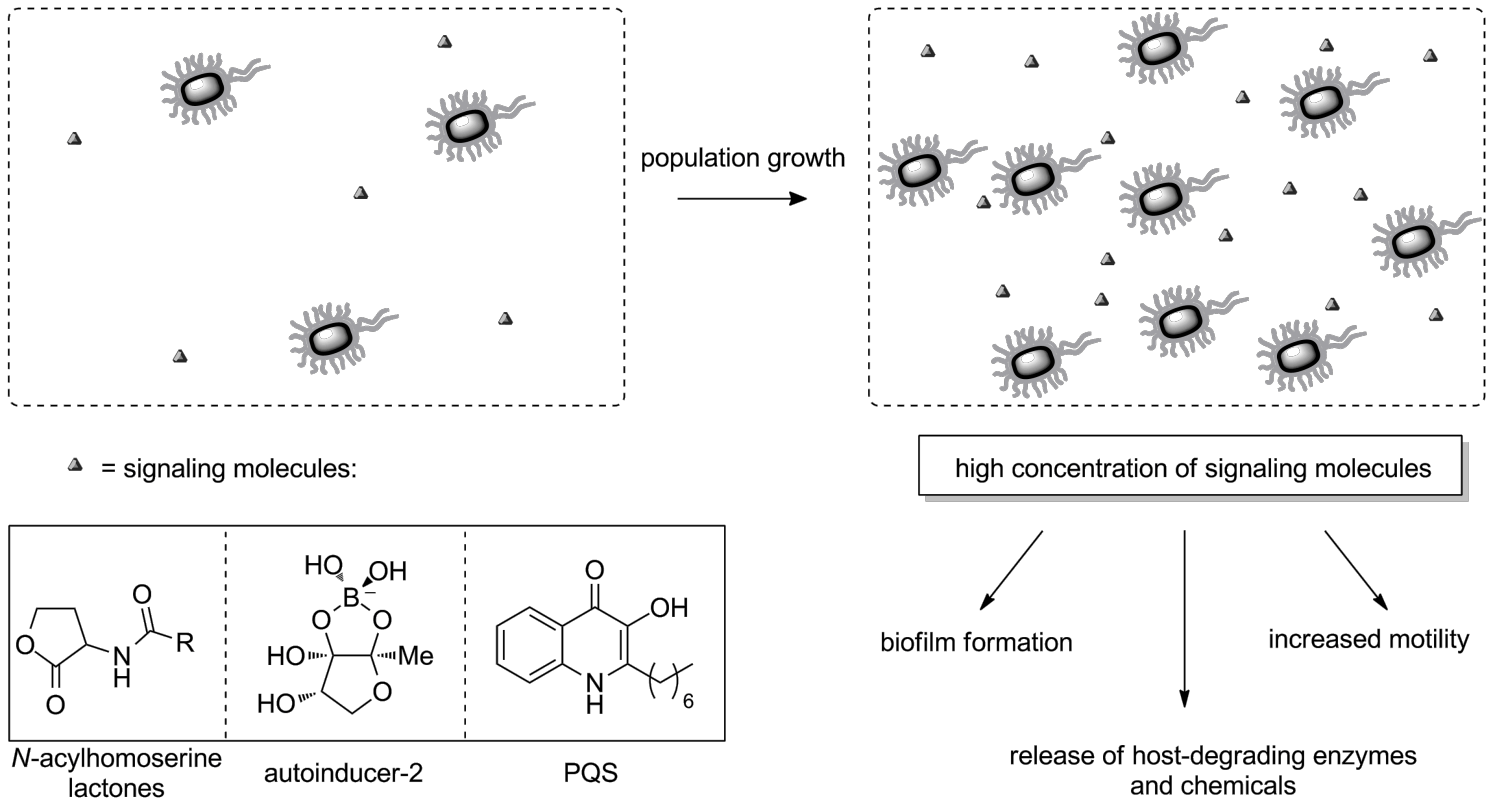
The mechanism of quorum sensing and representative signaling molecules.

Quorum sensing (QS) is employed by bacteria to communicate with each other in a given population [70]. In this regulatory mechanism, signal molecules (also known as autoinducers) are constantly secreted by each individual bacterium and at a defined population density the concentration of this molecular messenger reaches a threshold that activates quorum sensing-controlled processes (Figure 5). Many virulence traits are influenced by quorum sensing and thus developing methods to reduce virulence by interfering with bacterial communication is currently a topic of intense research efforts.

Quorum sensing exists in Gram-negative and Gram-positive bacteria. While Gram-positive bacteria often use peptides as signal molecules, Gram-negative bacteria employ *N*-acylhomoserine lactones (AHLs) with subtle differences in their chemical structure, as well as other types of autoinducers (Figure 5). Interestingly, the signaling molecule autoinducer-2 is used by both Gram-positive and Gram-negative species. Because highly structurally similar or even identical molecules are employed for bacterial signaling, it is obvious that bacteria also communicate between species, which can be of use for the bacterial community in co-infections. Often, multiple QS mechanisms exist within one species. For example in *P. aeruginosa*, four signaling systems have been identified to date, which are highly interconnected and mutually influence each other [30]. Some bacteria employ rather specific quorum sensing molecules, such as the Pseudomonas Quinolone Signal (PQS) and its biosynthetic precursors in *P. aeruginosa* some of which are also found in *Burkholderia* [71], two species that often co-infect patients for example in cystic fibrosis airways infections. By blocking QS processes, the release of virulence factors such as host degrading enzymes or chemicals, or the formation of bacterial biofilms, can be inhibited. Numerous reviews have detailed these processes addressing various QS pathways [30,72–73].

**Figure 6:**
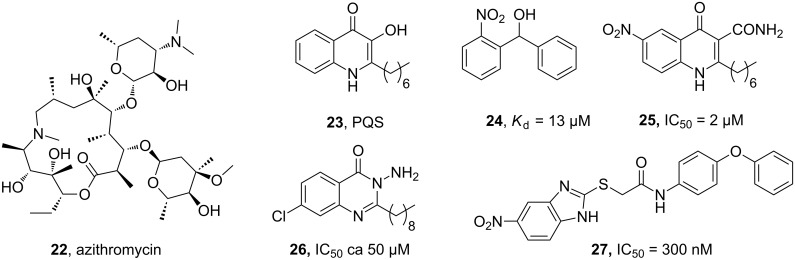
Inhibitors of bacterial quorum sensing.

The antibiotic azithromycin (**22**), which does not have significant bactericidal activity for *P. aeruginosa*, but interferes with its quorum sensing pathways, was studied in a clinical trial (Figure 6) [74]. The macrolide antibiotic of natural origin, which does not resemble the structures of signal molecules, was shown to reduce the presence of quorum sensing molecules in vitro and in vivo. It prevented the selection of QS-mutants (*lasR*) that rapidly appear in untreated patients and outgrow wild-type bacteria as a result of a fitness advantage. Thus, it may be of help in acute infections to reduce virulence, as stated by Köhler et al.

Many approaches towards developing QS antagonists as tool compounds and drug candidates start from the natural QS signal molecules and mimic their structures. The PQS system of *P. aeruginosa* is particularly attractive and can be considered as a pathogen specific target. The biosynthesis of the PQS signal **23** involves a set of biosynthetic enzymes PqsABCDEH and its autocatalytic receptor PqsR (MvfR). Biaryl methanols (e.g., **24**) function as PqsD transition state analogues, and were shown to inhibit the enzyme and reduce bacterial biofilm formation [75]. Numerous approaches target the signal molecule receptor PqsR, and compounds such as **25**, **26** and **27** successfully inhibited virulence factor production, biofilm formation and virulence in an insect infection model or a murine model [76–78].

## Conclusion

The current antimicrobial crisis poses an enormous challenge to society, and requires a joint effort for the development of novel anti-infectives. While there is an urgent need for new antibiotics with novel modes of action that avoid cross-resistance to established drug-resistant strains, the development of antivirulence drugs will address a promising new paradigm in antibacterial therapy, leading to a second anti-infective pillar.

It has to be emphasized that a concerted approach to new anti-infectives is of the utmost importance. Private and public research have to join forces to provide new treatments and to sustain a continuous supply of drugs with novel modes of action, necessary to maintain an arsenal that is able to treat MDR/XDR infections in the future. Some pathogens are well studied and numerous approaches have been developed, e.g., *P. aeruginosa* [30] and *S. aureus* [79–80]. It is, however, questionable why research on some of the most problematic pathogens discussed above, e.g., *Acinetobacter* or *Enterobacter*, is scarce and publications on their biology cover only a small fraction of the literature compared to the well-studied pathogens.

Adding to the prevalent resistance, recent reports [81–82] uncovered the abundance of various resistant pathogenic bacteria in proximity to antibiotic production facilities and their untreated sewage outlets in the Hyderabad area in India. One important factor for resistance development, also in industrialized countries, is the large scale exposure of organisms in the environment to released antibiotics. This can result from high drug concentrations in community waste water combined with ineffective drug clearance mechanisms, as well as from the excessive and inappropriate use of antibiotics in the commercial livestock breeding industry. Since numerous targets of classical antibiotics are conserved across a large number of bacterial species, it is obvious that resistance against antibiotics can also develop outside the patient in the environment. This process can take place in environmentally present pathogens, e.g., *P. aeruginosa*, but also in drug-exposed apathogenic bacteria, followed by horizontal transfer of the resistance gene into a pathogen, generating an uncontrollable risk for mankind. In contrast, it can be anticipated that resistance development of antivirulence compounds, which target specific mechanisms of the pathogen–host interplay, is absent outside a patient. Thus a reduced risk of resistance appearing both in the patient and in the environment would provide a benefit of antivirulence drugs over classical antibiotics.

It remains to be established whether antivirulence drugs will be sufficiently effective as a sole treatment, or if they will be used as adjuvants and co-application with antibiotics will be required. Antivirulence compounds dismantling biofilm-protected chronic pathogens or directly inhibiting bacterial factors of acute toxicity/virulence are likely to be successful as future therapies against the impending threat of highly antibiotic-resistant pathogens. In some cases, it was already shown that virulence blockers act synergistically in combination with antibiotics, for example against *P. aeruginosa* biofilms [44]. Therefore, it is likely that combinations of drugs will be applied for drug-resistant bacterial infections, as is currently the state of the art for many viral infections.
